# The Role of Cardiac Macrophage and Cytokines on Ventricular Arrhythmias

**DOI:** 10.3389/fphys.2020.01113

**Published:** 2020-09-23

**Authors:** Mingxian Chen, Xuping Li, Songyun Wang, Lilei Yu, Jianjun Tang, Shenghua Zhou

**Affiliations:** ^1^The Second Xiangya Hospital, Central South University, Changsha, China; ^2^Department of Cardiology, Renmin Hospital of Wuhan University, Wuhan, China

**Keywords:** macrophage, cytokines, electrophysiology, ventricular arrhythmias, connexin43

## Abstract

In the heart, cardiac macrophages have widespread biological functions, including roles in antigen presentation, phagocytosis, and immunoregulation, through the formation of diverse cytokines and growth factors; thus, these cells play an active role in tissue repair after heart injury. Recent clinical studies have indicated that macrophages or elevated inflammatory cytokines secreted by macrophages are closely related to ventricular arrhythmias (VAs). This review describes the role of macrophages and macrophage-secreted inflammatory cytokines in ventricular arrhythmogenesis.

## Origin and Functions of Cardiac Macrophages

Tissue-resident macrophages have been observed in various organs, including the heart, brain, liver, and lung, and originate from the embryonic lineage, which is different from that of monocytic progenitors (Ginhoux et al., 2010; Davies et al., 2013; Moore et al., 2013). Cardiac macrophages maintain a homeostatic population through their self-proliferative properties and are independent of blood monocyte-derived macrophages. Recently, in steady-state conditions, two resident cardiac macrophage subsets, MHC-IIlowCCR2- and MHC-IIhighCCR2- cells, were identified by gene fate-mapping techniques. Under cardiac injury conditions, a third cardiac macrophage population, MHC-IIhighCCR2+ cells, was identified in the heart (Epelman et al., 2014; Honold and Nahrendorf, 2014). Cardiac CCR2− macrophages originate from the primitive yolk sac and are replenished through local proliferation, whereas CCR2+ macrophages originate from bone marrow-derived monocytes and repopulate through monocyte recruitment and proliferation. Resident CCR2− macrophages are involved in angiogenesis and cardiomyocyte proliferation. Bajpai et al. (2018) demonstrated that the human myocardium is populated by distinct subsets of CCR2− macrophages, CCR2+ macrophages, and CCR2+ monocytes. Subsequently, Bajpai et al. (2019) showed that the depletion of resident cardiac CCR2- macrophages in a murine model of myocardial infarction increased the infarct area, reduced left ventricular (LV) systolic function, and aggravated LV remodeling. Interestingly, a recent study published in *Cell* demonstrated that resident macrophages in the steady-state heart facilitated electrical conduction, thus highlighting a novel concept regarding the potential role of cardiac macrophages in modulating cardiac electrical function (Hulsmans et al., 2017).

Under myocardial inflammatory conditions, monocyte-derived macrophages are recruited to the heart and characterized as MHC-IIhighCCR2+ cells. Mouse blood monocytes have been divided into Ly6C^*hi*^ and Ly6C^low^ (Robbins et al., 2012; Yap et al., 2019). Ly6C^*hi*^ monocytes induce excessive monocytosis, accumulate in the injured area, and differentiate into macrophages. Ly6C^low^ monocytes, derived from pro-inflammatory Ly6C^High^ cells, are less recruited than their Ly6C^High^ counterparts following MI. Ly6C^low^ monocytes are responsible for patrolling and tissue injury repair. However, human peripheral blood monocytes have 3 phenotypes: proinflammatory CD14^++^CD16^–^ monocytes, anti-inflammatory CD14^+^CD16^++^ monocytes with a function similar to Ly6C^low^ monocytes, and proinflammatory CD14^++^CD16^+^ monocytes secreting TNF-α (Heidt et al., 2014; van der Laan et al., 2014).

## Macrophage Activation Under Inflammatory Conditions

Macrophages are extremely heterogeneous and show adaptation of the phenotype and functions according to the surrounding microenvironment and aging (Gosselin et al., 2014; Pinto et al., 2014). Macrophage activation produces distinct functional phenotypes that are most commonly categorized as classically “inflammatory” macrophages and alternatively “anti-inflammatory” macrophages. This is a basic delineation of macrophage phenotypes but an overly simplistic view of macrophage behavior. Classically activated macrophages have proinflammatory properties, whereas alternatively activated macrophages are linked to cell proliferation and tissue repair (Nahrendorf et al., 2007; Biswas and Mantovani, 2010; Italiani and Boraschi, 2014). Classically activated macrophages secrete proinflammatory cytokines such as interferin-1β (IL-1β), IL-6, IL-12, tumor necrosis factor-α (TNF-α), and matrix metalloproteinase (MMP) and chemokines, which play a key role in host defense. Alternative activated macrophages produce cytokines, including transforming growth factor-β (TGF-β), IL-10, enzyme arginase-1 in mice (ARG 1) and chemokines, which are involved in collagen formation and tissue repair.

After myocardial infarction (MI), macrophages are abundant in the infarcted area. Ly-6C^High^ monocytes from the bone marrow and spleen are recruited to the infarcted zone and then differentiate into macrophages. The levels of this inflammatory subtype reach a peak approximately 3 days after injury (Heidt et al., 2014). Between days 5 and 7, macrophage populations are at their maximum within the infarct (Nian et al., 2004; Libby, 2013). Classically activated macrophages dominate the cell population of the infarcted zone. Classically activated macrophages produce proinflammatory cytokines, enhance the proinflammatory response and facilitate the breakdown of collagen. The reparative phase is characterized by phenotypic transition from inflammatory monocytes and macrophages (Ly-6C^High^ monocytes and classically activated macrophages) to the anti-inflammatory subtypes (Ly-6C^low^ monocytes and alternatively activated macrophages) (Nahrendorf et al., 2007). The pool of cardiac macrophages is replenished as Ly-6C^low^ monocytes are extensively recruited to the infarcted area. Ly-6C^low^ monocytes mainly derive from pro-inflammatory Ly-6C^High^ monocytes (Hilgendorf et al., 2014). After accumulation, Ly-6C^low^ monocytes are thereby differentiated into alternatively activated macrophages. Alternatively activated macrophages release IL-10, which inhibits the proinflammatory effects of classically activated macrophages, and TGF-β, which promotes tissue remodeling and angiogenesis (Lavine et al., 2014). Fei et al. (2019) demonstrated that activated macrophages directly connected to cardiomyocytes, thereby prolonging the action potential duration (APD), and ultimately led to APD heterogeneity and post-MI arrhythmias via gap junctions. This finding suggests that macrophages directly participate in ventricular arrhythmias after myocardial injury.

## Macrophage-Related Inflammation and Ventricular Arrhythmias

Previously, several clinical studies provided evidence that increased inflammatory cytokines are closely associated with cardiac arrhythmias (Lewek et al., 2014). These results suggested that inflammation affects the initiation and progression of VAs. The proarrhythmic effects involve substrate-triggered cardiac electrical and structural remodeling. Inflammation contributes to the occurrence of ectopic-triggered activity and re-entry (Wakili et al., 2011; Dobrev et al., 2012; Aulin et al., 2015).

Electrophysiological changes (ion channel disturbance, early and late afterdepolarizations), as well as gap junction remodeling and enhanced myocardial fibrosis, are immune-related mechanisms responsible for cardiac arrhythmias. Macrophage-dependent and macrophage-independent inflammation, including cytokine processes, serves as the basis of proinflammatory-induced VAs. Inflammation in the heart can also directly result in fluctuations in membrane potential. VAs can be triggered by early afterdepolarizations (EADs) and delayed afterdepolarizations (DADs). EAD results from the reduced function of potassium channels or the increased function of calcium or sodium channels. Abnormal intracellular Ca^2+^ handling, such as sarcoplasmic reticulum (SR) overload and uncontrolled Ca^2+^ leak, contributes to DAD (Kao et al., 2010; Chilukoti et al., 2013). Furthermore, gap junctions (GPs) are cell-to-cell pathways mediating electrical and chemical signal exchange between adjacent myocytes. GPs can transmit an orderly wave of electrical excitation. In the ventricles, gap junction remodeling, including connexin43 (Cx43) reduction, Cx43 dephosphorylation and Cx43 lateralization in pathological conditions, produces arrhythmia substrates (Duffy, 2012). The inflammatory response in the local heart area may play a crucial role in GP remodeling. Finally, excessive fibrosis or cardiac sarcoid infiltrated with abundant macrophages produces VAs not only by the mechanism of triggered substrate but also by re-entry (Okada et al., 2018). On the one hand, Haider et al. (2019) showed that cardiac macrophages can develop a fibroblast-like phenotype and directly contribute to the formation of fibrosis after myocardial infarction. On the other hand, macrophage-derived cytokines activate fibroblasts and produce cardiac fibrosis (Jung et al., 2017; Shimodaira et al., 2018; Abe et al., 2019). Cardiac fibrosis complicates electrical impulse propagation, slows conduction velocity, and forms unidirectional conduction blocks (Rohr et al., 1997; Rohr, 2012). Inflammation is also related to tissue repair after injury. Tissue repair is accompanied by parenchymal cell regeneration and finally fibrous tissue formation, namely, scar formation (Klein et al., 2000; Said et al., 2011; Gorenek et al., 2014; Vonderlin et al., 2019).

## Macrophage-Induced Cardiac Sympathetic Remodeling and Ventricular Arrhythmias

Sympathetic overactivity and structural remodeling play a critical role in ventricular arrhythmogenesis (Podrid et al., 1990; Shen and Zipes, 2014; Kalla et al., 2016). In an experimental study, sympathetic nerve stimulation caused a change in ventricular electrophysiology, reduced the ventricular fibrillation threshold and triggered Vas (Mantravadi et al., 2007; Ng et al., 2009). Although it is difficult to induce VAs in mice, norepinephrine (NE) injected into the epicardial tissue of guinea pigs elicits triggered automaticity, and computational modeling identified a Ca^2+^ overload mechanism in cardiac ventricular electrophysiology. This finding supports the hypothesis that heterogeneity or gradients of sympathetic activation are proarrhythmic (Vaseghi et al., 2012). A growing body of work has demonstrated that macrophages can contribute to sympathetic hyperactivity. Levick et al. (2010) showed that substance P released by sympathetic afferent fibers could bind to the neurokinin-1 receptor of macrophages to induce the production of macrophage-derived angiotensin II. Angiotensin II could further stimulate the terminus of sympathetic efferent fibers and then increase the production of norepinephrine. Thus, macrophages play a critical role in mediating VAs relevant to sympathetic activity by enhancing the production of angiotensin II (Levick et al., 2010). Furthermore, peripheral proinflammatory factors produced by macrophages, such as IL-1β, IL-6, and TNF-α, can transmit signals to the brain by the circulation or through afferent fibers, which in turn activate the sympathetic nervous system (SNS).

A proarrhythmic substrate is usually formed by regional myocardial remodeling, as well as heterogeneity of sympathetic innervation (Barber et al., 1983; Tomaselli and Zipes, 2004). The heterogeneity of sympathetic innervation is called nerve sprouting (Cao et al., 2000; Chen et al., 2001). Emerging data have indicated that cardiac sympathetic sprouting and cardiac electrical remodeling are involved in the post-MI remodeling process (Zipes and Rubart, 2006; Wang et al., 2012). Sympathetic nerve sprouting may produce electrical and structural remodeling following AMI, resulting in electrophysiological instabilities and finally inducing VAs. In fact, studies have shown that the inhibition of sympathetic nerve sprouting induced by MI can exert antiarrhythmogenic effects (Wernli et al., 2009; Yang et al., 2016; Yin et al., 2016; Hu et al., 2019). Sympathetic nerve remodeling is a complex pathophysiological process, and sympathetic nerve sprouting is closely associated with the inflammatory reaction and is primarily present at the infarct border zone, where abundant macrophages and macrophage-derived cytokines are observed. Macrophages could promote sympathetic hyperinnervation via the regulation of nerve growth factor (NGF) expression (Yin et al., 2016). Atorvastatin could effectively improve cardiac sympathetic nerve remodeling by modulating macrophage polarization (Yang et al., 2016). It was recently demonstrated that inhibiting miR-155 can downregulate NGF expression by decreasing M1 macrophage polarization, subsequently impairing sympathetic nerve remodeling and VAs induced by acute myocardial infarction (Wernli et al., 2009; Hu et al., 2019).

Sympathetic nervous system can affect macrophages at both the systemic and regional levels (Chen et al., 2016). SNS fibers innervate the primary and second lymphoid organs (bone marrow, thymus, spleen, and lymph nodes) and are capable of modulating immune functions. The SNS contributes to the differentiation, maturation, recruitment, and regulation of macrophages. Moreover, the SNS directly innervates the target lesion, plays a proinflammatory role and promotes M1 polarization. Norepinephrine released from sympathetic nerve endings could bind to α- or β-adrenergic receptors expressed on immune cells (T cells, B cells, natural killer cells, and macrophages). This response gives rise to a cascade of events, including the production of proinflammatory cytokines and recruitment of leukocytes. Therefore, these changes might form a vicious circle between SNS activity and M1 polarization, both contributing to ventricular arrhythmogenesis.

## Cytokines Secreted by Macrophage and Ventricular Arrhythmias

**FIGURE 1:**
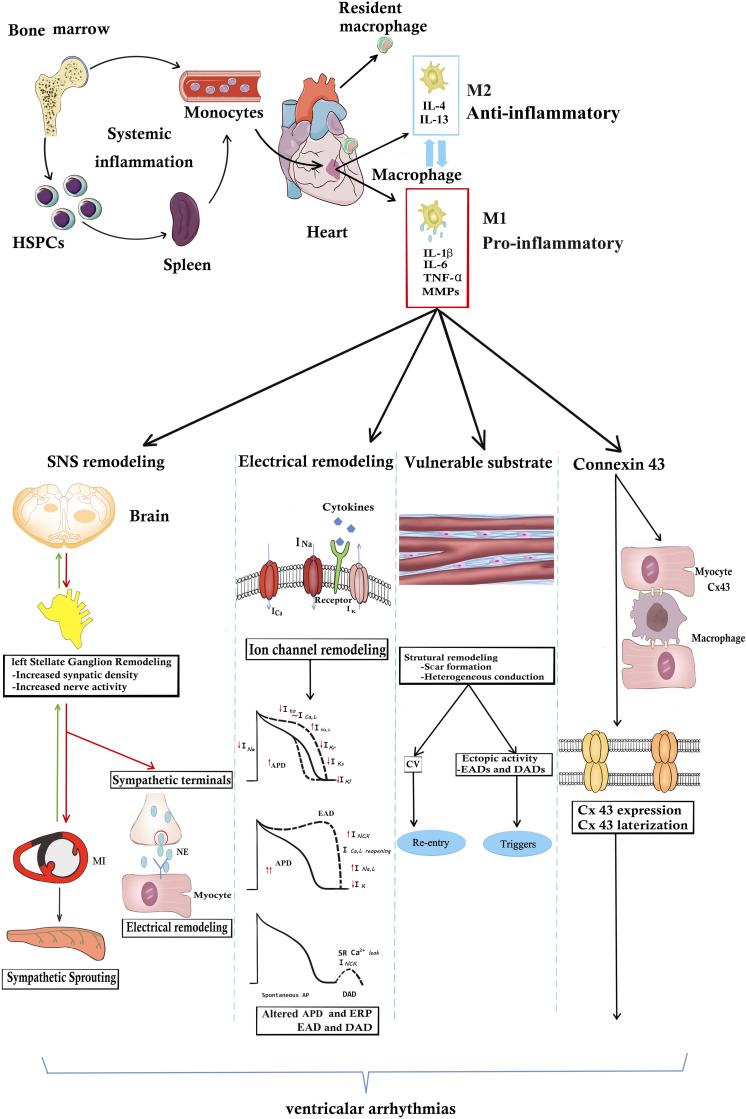
The underlying mechanisms of macrophages or macrophage-related cytokines produce VAs. In the steady state, resident cardiac macrophages originate from the yolk sac and fetal liver progenitors. Following myocardial inflammation, blood monocytes derives from increased production in the bone marrow and spleen. Monocyte-derived macrophages generally are divided into M1 “inflammatory” macrophages and “anti-inflammatory” M2 macrophages. M1 macrophages secrete pro-inflammatory cytokines including IL-1β, TNF-α, IL-6, and MMP-9. Macrophages or macrophage-related cytokines induce the occurrence of VAs through the following four pathways. (1) Inflammation increases afferent nerve traffic and then leads to anatomic remodeling within the left stellates ganglion remodeling and sympathetic sprouting. NE released from sympathetic terminals changes ventricular electrophysiology. SNS remodeling and over-activated sympathetic tone increase the propensity for cardiac VAs. However, in turn sympathetic tone aggravates macrophage activation. (2) Arrhythmogenic electrophysiological remodeling. Cytokines bind to the receptors and lead to ion channel remodeling, finally resulting in APD prolongation and ERP shorting. Altered ionic currents contribute to EAD and DAD increasing the arrhythmogenic substrate. (3) Macrophage-related cytokines induce the scar formation and CV heterogeneity, resulting in re-entry. Structural remodeling also induces EDA and DAD, producing arrhythmogenic triggers. (4) Inflammation directly changes the Cx43 structural and functional remodeling. However, whether inflammation promotes Cx43 remodeling between myocytes and macrophages still remain unknown. It needs further study. APD, action potential; Cx43, connexin43; CV, conduction velocity; DAD, delayed afterdepolarization; EDA, early afterdepolarization; IL-1β, HSPCs, hematopoiesis precursor cells; interleukin 1β; IL-6, interleukin 6; MMP-9, matrix metalloproteinases 9; NE, norepinephrine; TNF-α, tumor necrosis factor-α.

Macrophages secrete multiple cytokines. Proinflammatory factors produced by macrophages, such as IL-1β, IL-6, TNF-α, and matrix metalloproteins (MMPs), can regulate cardiac SNS activity, form a proarrhythmic substrate and directly affect myocardial electrophysiology (Hirayama et al., 2017). VAs might be induced by cytokines derived from macrophages in both acute and chronic diseases. It was shown that cytokines derived from macrophages following acute MI could target cardiac myocytes and induce electrophysiological remodeling, including a reduction in repolarizing K^+^ currents, Cx43 expression and intracellular Ca^2+^ mishandling (Pinto and Boyden, 1999; Francis Stuart et al., 2016). These changes could provide the trigger and substrate for ventricular arrhythmias (De Jesus et al., 2017). In the case of chronic inflammation, a growing body of clinical evidence has demonstrated that the serum concentration of macrophage-derived cytokines was significantly higher in post-MI patients with ventricular arrhythmias than in post-MI patients without ventricular arrhythmias (Streitner et al., 2007, 2009; Francis Stuart et al., 2016). Furthermore, Monnerat et al. (2016) experimentally demonstrated that IL-β derived from cardiac macrophages could trigger ventricular arrhythmias in mice. Even in the absence of cardiac injury, systemic inflammation was found to be related to an increased risk for ventricular arrhythmias. Extracardiac injury was also shown to enhance macrophage-related inflammation in the heart. VAs are also one of the most critical complications after renal or brain injury. Renal ischemia reperfusion increased susceptibility to ventricular arrhythmias depending on activation of NLRP3-CASP1-IL-1β; accordingly, this effect was inhibited by macrophage depletion (Alarcon et al., 2019). Rheumatoid arthritis is a chronic inflammatory disease, and increased serum concentrations of macrophage-derived cytokines were detected. Increased susceptibility to VAs was also found in patients with rheumatoid arthritis (Lazzerini et al., 2017a). The role of individual cytokines on VAs is listed below in detail (Figure 1).

IL-1β contributes to electrical function not only through direct effects but also indirect effects on VAs. Wang et al. confirmed that IL-1β injection into the left stellate ganglion (LSG) could increase sympathetic activity and the occurrence of VA. IL-1β injection induced cardiac electrical remodeling, and this response was attenuated by IL-1Ra preinjection (Wang et al., 2017). Cardiac fibrotic substrate and Cx43 remodeling are important mediators responsible for the heterogeneity in ventricular conduction for reentry. Considerable experimental data have shown that excess fibrosis promotes ectopic triggers in the hearts of aged rats and rabbits (Bapat et al., 2012; Wang et al., 2017). This kind of proarrhythmic substrate produced early afterdepolarizations, triggered activity and reduced conduction velocity (Bapat et al., 2012). Previous study has shown that IL-1β plays an important role in the formation of fibrotic substrates and is implicated in Cx43 remodeling, induced cell–cell uncoupling, lateralization, and degradation (Baum et al., 2012).

Thus, macrophage-derived IL-1β during myocardial healing could induce deleterious electrophysiological consequences.

### IL-1β and Ventricular Arrhythmias

IL-1, as an activating factor of endothelial cells, could regulate and initiate inflammatory responses. The IL-1 family is a group of 11 cytokines. IL-1α and IL-1β are the most studied members because of their early discovery and significant proinflammatory effects. IL-1β is synthesized as a precursor protein after stimulation by activated monocyte-macrophages (Guillén et al., 1995; Saxena et al., 2013).

IL-1β is a crucial regulator in the inflammatory response after MI and is involved in the modulation of immune cell recruitment, cytokine production, and extracellular matrix turnover. Clinical studies have revealed that the levels of IL-1β in both tissue and plasma are significantly increased in patients with VAs. These results suggest that in addition to the above biological functions, IL-1β might be an important mediator of ion channel remodeling, thereby producing Vas (Fernández-Sada et al., 2017; Abbate et al., 2020).

Emerging evidence has demonstrated that IL-1β can directly affect the electrical properties of cardiomyocytes (CMs). IL-1β resulted in changes in Ca^2+^ handling (Alarcon et al., 2019). Li and Rozanski (1993) found an increase in I*_*CaL*_* in guinea pig myocytes, inducing the prolongation of the action potential duration (APD) and the effective refractory period (ERP). Moreover, Liu et al. observed that IL-1β decreased the responsiveness of I*_*CaL*_* to β-adrenergic stimulation (Liu et al., 1999). IL-1β, synergistic with TNF-α application, also affected SR Ca^2+^ release and reuptake in rat ventricular myocytes, which contributed to the depressed Ca^2+^ transient and contractility (Duncan et al., 2010). Spontaneous SR Ca^2+^ release may increase the susceptibility to arrhythmias, leading to cell depolarization. Recently, Monnerat et al. (2016) showed that IL-1β produced by macrophages derived from the hearts of individuals with diabetes mellitus could directly target cardiomyocytes to induce VAs. IL-1β then induces a decrease in the Ito current and an increase in Ca^2+^ sparks, resulting in increased electrical vulnerability to arrhythmias. In a mouse model, De Jesus et al. (2017) found that IL-1β inhibition improved conduction velocity, reduced APD dispersion, improved intracellular Ca^2+^ handling, decreased the transmembrane potential and the magnitude of the Ca^2+^ alternans, and thus reduced spontaneous and inducible Vas (Su et al., 2018).

### TNF-α and Ventricular Arrhythmias

TNF-α is one of the most important inflammatory factors and is mainly secreted by activated macrophages. Myocardial TNF-α expression was significantly upregulated post AMI (Feldman et al., 2000). TNF-α has widespread biological effects on cell proliferation, differentiation, apoptosis and inflammatory reactions (Libby et al., 2002; MacEwan, 2002). Clinical evidence confirmed that the elevation of plasma TNF-α in patients with AMI was closely related to the occurrence of Vas (Halawa et al., 1999; Eskandarian et al., 2013). Experimental studies also showed that transgenic animals with TNF-α overexpression are prone to severe Vas (London et al., 2003; Chen et al., 2010).

TNF-α may alter the electric activity of cardiac myocytes by different mechanisms and finally induce Vas (Petkova-Kirova et al., 2006). TNF-α modulates cardiac *K*^+^ channels. TNF-α can induce a significant reduction in Ito density, modify Ito inactivation, and downregulate *K*_*v4.2*_ protein expression. TNF-α could inhibit the cardiac delayed rectifier K current via the protein kinase A (PKA) pathway (Hatada et al., 2006). Furthermore, TNF-α appears to have a significant impact on cellular Ca^2+^ release and uptake. This molecule disrupted cellular *Ca*^2+^ cycling, which increased the probability of proarrhythmic spontaneous *Ca*^2+^ release from the SR, which may contribute to the increased incidence of arrhythmia in sepsis in isolated rat ventricular myocytes (Duncan et al., 2010). It was found that the regulation of *Ca*^2+^ inflow of cardiac myocytes was achieved by the phospholipase A2/arachidonic acid (PLA2/AA) pathway (Amadou et al., 2002).

Slowed myocardial conduction velocity (CV) increases the risk of re-entrant excitation, predisposing patients to cardiac arrhythmia. CV is determined by the ion channel and cellular interconnections. George et al. (2017) demonstrated that TNFα could reduce CV by altering electrical coupling between myocytes in guinea pig hearts. The effects of TNF-α on gap junction coupling have been extensively studied. TNF-α alters Cx43 expression, reduces Cx43 phosphorylation, and alters Cx43 redistribution, which is important in modulating Cx43 channel conductance (Fernandez-Cobo et al., 1999; Sawaya et al., 2007; George et al., 2017).

### IL-6 and Ventricular Arrhythmias

IL-6 is also involved in multiple biological effects, including cardiomyocyte response to injury (Yang et al., 2004; Alí et al., 2018). Serum levels of IL-6 and its mRNA and protein expression in cardiac tissues are significantly increased in patients with cardiac diseases, including heart failure, myocarditis, septic cardiomyopathy, myocardial infarction, and cardiac myxoma (Ikeda et al., 1992). IL-6 plays a critical role in the pathophysiology of these cardiac disorders. A study demonstrated that elevated serum IL-6 levels were associated with an increase in susceptibility to spontaneous ventricular tachyarrhythmia in patients with coronary artery disease (Streitner et al., 2007). Recently, accumulating data obtained from patients with myocarditis/endocarditis and systemic autoimmune diseases (Ukena et al., 2011), particularly rheumatoid arthritis (Lazzerini et al., 2015a) and other connective tissue diseases, demonstrated that circulating IL-6 levels are elevated in these patients. Increased IL-6 levels are correlated with vulnerability to QT interval prolongation, which contributes prominently to arrhythmic events and torsade de pointes (TdP) (Adlan et al., 2015; Lazzerini et al., 2015b; Lazzerini et al., 2017b). A study also indicated that IL-6 possessed a potential direct electrophysiological effect on ion channels that can alter the APD and QTc interval (Aromolaran et al., 2018).

Emerging experimental evidence showed that IL-6 could regulate the electrophysiological properties of cardiomyocytes. Previous data suggest that IL-6 may play a critical role in contributing to the modulation of *I*_*Ca,L*_ and *I*_*K*_ currents, and both factors are active contributors to cardiac instabilities. Hagiwara et al. (2007) found that IL-6 could regulate I*_*CaL*_* and density. After acute (30 min) exposure to IL-6 and soluble IL-6 receptor (IL-6R), the I*_*Ca,L*_* density in mouse ventricular myocytes was significantly increased, which was strongly associated with LQTS. IL-6 was also shown to cause QT prolongation by suppressing *I*_*Kr*_. Ademuyiwa et al. demonstrated that IL-6 alone or in combination with soluble IL-6R could inhibit the *I*_*Kr*_ peak and result in the prolongation of APD via Janus kinase (JAK) pathway activation, forming the basis for the observed clinical QT interval prolongation (Aromolaran et al., 2018). *In vitro* studies have shown that IL-6 suppresses peak cytosolic intracellular Ca^2+^ and cell contraction of cardiomyocytes within minutes due to activation of Ca^2+^-dependent nitric oxide synthetase (Kinugawa et al., 1994; Yu et al., 2003; Monnerat et al., 2016).

### MMP-9 and Ventricular Arrhythmias

In hearts, myocardial injury was shown to activate macrophages to increase MMP-9 secretion. Clinical data have shown that serum MMP-9 levels are significantly elevated in patients with cardiac dysfunction (Li et al., 1998; Thomas et al., 1998; Sivakumar et al., 2008) and are closely associated with increased VAs and sudden cardiac death (Flevari et al., 2012; Hästbacka et al., 2012; Turkdogan et al., 2017), while the downregulation of MMP-9 by gene modification or pharmacological inhibition significantly reduced the incidence of VAs in a mouse model (Weng et al., 2016). The above evidence indicates that MMP-9 plays a critical role in the pathophysiology of VAs.

Experimental data demonstrated that MMP-9 was mainly involved in the regulation of cardiomyocyte electrophysiological properties via the formation of cardiac fibrosis, gap junction remodeling and calcium homeostasis (Weng et al., 2016). Cardiac fibrosis, Cx43 reduction and lateralization are prerequisites of ventricular conduction heterogeneity for re-entry. MMP-9 is a key regulator of the reparative phases of post-MI healing and a necessary modulator for proper scar formation. MMP-9 could enhance myocardial remodeling, result in excessive extracellular matrix degradation, increase myocardial fibrosis, and thus contribute to re-entry and eventually lead to VAs. MMP-9 might also degrade Cx43, which is required for proper cell–cell electrical coupling (Fontes et al., 2012; Nguyen et al., 2014). Mukherjee et al. (2010) demonstrated that MMP-9 activity corresponded to increased Cx43 lateralization and reduced conduction velocity. Excessive MMP-9 could disrupt normal cell–cell electrical communication post-MI. A recent study showed that MMP-9 could increase Ca^2+^ leakage from the SR, which could depolarize cardiomyocytes and trigger fatal arrhythmia. MMP-9 also decreases CD36 and increases PKA activity. Activated PKA subsequently triggers ryanodine receptor 2 (RyR2) phosphorylation, leading to a higher probability of RyR pore opening, followed by increased calcium leakage. An increase in calcium sparks activates an arrhythmogenic depolarizing inward Na^+^/Ca^2+^ exchange current, which causes delayed afterdepolarizations and triggers VAs and sudden cardiac death (Marx et al., 2000; Dobrev and Wehrens, 2014; Wehrens et al., 2004; DeLeon-Pennell et al., 2016).

## The Connexins Between Macrophages and Cardiomyocytes on Ventricular Arrhythmias

In addition to antigen presentation, phagocytosis and immunomodulation, cardiac macrophages have been proven to directly induce cardiac electrophysiological changes. This finding substantially changed our previous knowledge of macrophage function. Previous studies have shown that the transmembrane potential is −26 mV for mouse macrophages and −18 mV for guinea pig macrophages (Dos Reis et al., 1979). Macrophages present several different types of K^+^ and Cl^–^ channels in the membrane. Membrane potential and ionic conductance regulate many cell functions, including transmembrane signaling, phagocytosis, secretion, and motility (Udagawa, 2003; Pinto, 2017). However, previous data did not show that macrophages could directly modulate cardiac electrophysiology.

A recent study reported by Hulsmans et al. (2017) demonstrated that cardiac resident macrophages are integral for normal heart rhythm. These researchers found that cardiac macrophages are highly abundant at the atrioventricular node (AVN) in mice and humans. They found that macrophages connect to cardiomyocytes at the AVN via Cx43, forming punctate junctions between macrophages and cardiomyocytes. Cx43-mediated macrophage-CM coupling served as electrical coupling of the two cell types. In an *in vitro* study, cocultures of macrophages and mouse neonatal AVN CMs also established Cx43 coupling between the two cell types. The researchers further showed that depolarization of macrophages improved AVN conductance. However, depletion of macrophages resulted in AVN block, resulting in arrhythmia.

In the injured heart, the number of cardiac macrophages is significantly increased compared with that in the uninjured heart. Considering that resident macrophages might participate in cardiac electrophysiology through Cx43 between macrophages and myocytes, it would be interesting to investigate whether monocyte-derived macrophages recruited to the myocardium under inflammatory conditions also connect to myocytes by Cx43, affecting cardiomyocyte electrophysiology and resulting in VAs. Fei et al. (2019) found that proinflammatory macrophages formed gap junctions with cardiomyocytes and accumulated in MI border zones 3 days post-MI. These researchers further demonstrated that non-inflammatory macrophages connected with myocardiocytes could shorten APD_90_, yet proinflammatory macrophages could prolong APD_90_ in an *in vitro* coculture experiment. This finding indicates that the Cx43 connection between macrophages and cardiomyocytes leads to APD heterogeneity and post-MI arrhythmias. Therefore, targeting macrophage-CM coupling could be a potential useful target when treating inflammation-associated conduction abnormalities.

## Future Therapeutic Direction and Conclusion

Macrophages appear to play direct and indirect roles in the occurrence of VAs. Activation of cardiac macrophages induces VAs through sympathetic nerve sprouting, proinflammatory cytokine production, and the direct influence of cardiac electrophysiology. Previously, inflammation has always been treated as an epiphenomenon and not suitable as a target for intervention. However, based on experimental and clinical evidence, anti-inflammatory therapy targeting the inflammatory cytokines contributed to a benefit from cardiovascular diseases (Ridker et al., 2017; Wang et al., 2017). Canakinumab Anti-inflammatory Thrombosis Outcomes Study (CANTOS) has been provided the evidence that IL-1β treated as a target and reduced major cardiovascular events. It opens a landscape of cardiovascular diseases therapy (Ridker et al., 2017). Therefore, immunotherapy for therapeutic interventions, targeting these functional elements of macrophages and cytokines blockade, are likely to be a promising new research avenue and might be valuable for the purpose of developing new therapeutics to reduce VAs.

## Author Contributions

MC, XL, and SW participated in the study design and drafted the manuscript. SZ and MC responsible for writing the manuscript. LY, JT, and SZ participated in the overall editing and approval of the manuscript. All authors contributed to the article and approved the submitted version.

## Conflict of Interest

The authors declare that the research was conducted in the absence of any commercial or financial relationships that could be construed as a potential conflict of interest.
